# The Evaluation on the Credit Risk of Enterprises with the CNN-LSTM-ATT Model

**DOI:** 10.1155/2022/6826573

**Published:** 2022-09-22

**Authors:** Lei Zhang

**Affiliations:** ^1^School of Mathematics and Statistics, Chongqing Jiaotong University, Chongqing 400074, China; ^2^School of Economics and Management, Chongqing Jiaotong University, Chongqing 400074, China

## Abstract

Credit evaluation is a difficult problem in the process of financing and loan for small and medium-sized enterprises. Due to the high dimension and nonlinearity of enterprise behavior data, traditional logistic regression (LR), random forest (RF), and other methods, when the feature space is very large, it is easy to show low accuracy and lack of robustness. However, recurrent neural network (RNN) will have a serious gradient disappearance problem under long sequence training. This paper proposes a compound neural network model based on the attention mechanism to meet the needs of enterprise credit evaluation. The convolutional neural network (CNN) and the long short-term memory (LSTM) network were used to establish the model, using soft attention, the gradient propagates back to other parts of the model through the attention mechanism module. In the multimodel comparison experiment and three different enterprise data experiments, the CNN-LSTM-ATT model proposed in this paper is superior to the traditional models LR, RF, CNN, LSTM, and CNN-LSTM in most cases. The experimental results under multimodel comparison reflect the higher accuracy of the model, and the group test reflects the higher robustness of the model.

## 1. Introduction

Small and medium-sized enterprises (SMES) are playing an increasingly important role in supporting the national economy and have made great contributions to innovative output and labor employment. However, due to the difficulties of credit evaluation, small and medium-sized enterprises are currently facing the problem of financing and loans, which has great resistance to the operation and development of enterprises. In this context, it is urgent to build a reliable credit and risk assessment system to solve the problem of the lack of credit and risk assessment in the financing and loan process of small and medium-sized enterprises, and this is of great significance for financial services supervision.

In recent years, most credit risk evaluation models have been paid much attention by many scholars, which are constructed by statistical and machine learning methods [1–9]. The traditional methods such as LR and RF have low accuracy when the feature space is large. Meanwhile, CNN is widely used in computer vision simulation [10, 11] and human speech recognition [12–14]. Due to the explosive development of big data, which drives the development of neural networks, research results in related fields of CNN have achieved considerable progress in a wide range of classification and regression [15, 16]. The problem of serious gradient loss in RNN, LSTM, and neural network has also been developed rapidly in the field of emotion classification under a long sequence [17–20]. With the development of the economy and the development of neural networks, it is necessary to combine neural networks and risk evaluation issues. At the same time, the model combining CNN and LSTM has been applied to text classification [21–24], image recognition [25–28], emotion analysis [21, 29, 30], and other fields. Based on the deep learning method of CNN and LSTM, this paper aims at studying the problem of the enterprise's credit score by using enterprise's behavior data. Based on the enterprise's behavior data, the proposed model used in this paper integrates CNN and LSTM to evaluate the credit risk of enterprises. LSTM could act in cooperation for the information back and forth in the long sequence, which has the better performance than the general recurrent neural network in the long sequence training [31, 32]. CNN could extract vertical and horizontal features. In this paper, CNN is used to extract features of the behavioral matrix. Moreover, attention mechanism is added to the model to extract important features [33–35]. In the aspect of data processing, the behaviors of the enterprises are represented by the behavior matrix, which could improve the predictive effect and understandability of the model.

Motivated by the above analysis, the CNN-LSTM-ATT composite neural network model is proposed to solve the problem of enterprises credit risk evaluation. The selection experiment for hyperparameter and comparison experiment among different models are conducted on the realistic data set. The accuracy and robustness of the CNN-LSTM-ATT model in solving the problem of credit risk evaluation are comprehensively analyzed. Based on the above discussion, this paper aims at solving the following problems:

- First of all, the financial information and behavior data of local enterprises are conducted, and data coding is represented as the two-dimensional matrix. After data preprocessing, the variable data set could finally be used as the input of neural networks, which have better effects on the training and testing of the model. There will be a lot of missing values and unqualified values in the original big data set. The missing values, that are some data of the enterprises may not exist or cannot be filled. For such data, we will fill them by comparing the average value of the whole data of enterprises. Unqualified values are that wrong values and abnormal outliers during the inputting process and are disposed by complementing the default values. After data processing, the data set could improve the accuracy and efficiency of prediction.
- Secondly, the CNN-LSTM model is used for feature extraction of enterprise's behavior data in this paper. CNN model is used for feature extraction of enterprises, which is manifested in enterprise's behavior data. LSTM model is used for feature extraction under the long sequence of enterprises, which could prevent the occurrence that the effect of debt during earlier stage on the normal loan behavior after the later development.
- Thirdly, the parameters of CNN, LSTM, and attention mechanism are adjusted to make sure that the predicted result could reach the expected acceptable result. In the neural network, the influence of parameters on the model results is especially obvious, which has a great influence on the prediction result. The parameters of all models in this paper need to be adjusted to the most reasonable conditions to achieve the best forecast results.
- Finally, the comparison among the traditional single CNN model, single LSTM model, and proposed CNN-LSTM-ATT are carried out by using the control variable method. The prediction effect of the model is verified by calculating the AUC value. And the realistic enterprise's behavior data are substituted into the proposed model to verify the realistic environment. Therefore, it could achieve the correct prediction result.

## 2. Model Description and Preliminaries

### 2.1. Long Short Term Memory

Long short-term memory is used for feature extraction under the long sequence of enterprises. LSTM could act in cooperation for the information back and forth in the long sequence, which has the better performance than the general recurrent neural network. LSTM could deal with the explosive gradient and the vanishing gradient in the long sequence training.

**Figure 1:**
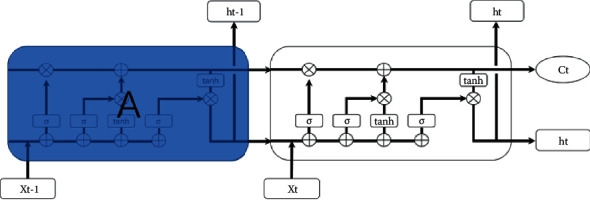
The unit structure of LSTM.

LSTM has designed a special structural unit and three unique “gate” structures. This structure can selectively increase or remove the information of passing through the unit. So as to screen the information passing through the unit. The “gate” structure is implemented by Sigmoid function and the Sigmoid value ranges from 0 to 1, which can be regarded as how much information is allowed to pass through. The closer this value is to 0, the more difficult it is in passing through the information, and the more likely it will be abandoned. On the contrary, the closer the value is to 1, the less difficult it is in passing through the information, and the less likely it will be abandoned. The hidden layer of LSTM is formed by the following structure, as shown in Figure 1.

The structure includes the input *x*_*t*_, the state variable *c*_*t*_, the temporary state variable ${\tilde{c}}_{t}$, the hidden layer state *h*_*t*_, the forgotten gate *f*_*t*_, the memorial gate *i*_*t*_, and the output *o*_*t*_.

### 2.2. Convolutional Neural Networks

Convolutional neural networks is a kind of special feedforward neural networks containing convolution computation. It occupies a powerful position in deep learning. In terms of image recognition and human language analysis, CNN has the ability of representation learning, which enables CNN to classify translational invariant on input information according to its own hierarchical structure.

The general neural network structure is composed of the input layer, the hidden layer, and the output layer. The hidden layer of CNN has three different structures from the general NN model: the convolutional layer, the pooling layer, and the fully connected layer. In some more modern algorithms, there may be inception block, residual block, and other complex constructions. In the process of constructing neural networks, the convolutional layer and the pooling layer are unique for the convolutional neural networks.

### 2.3. Attention Mechanism

The concept of attention mechanism is from the research of human vision and related fields by experts and scholars. Attention mechanism realizes the rapid and effective allocation of resource information processing. In essence, a weight of attention mechanism is added to the hidden layer of the neural network to capture the key features of information. It is found that the importance of feature saliency is positively correlated with the amount of contained information. In other words, the greater the importance of feature saliency, the more information will be contained, which will have a greater influence on the actual demand.

The channel attention mechanism learns the weight of each channel in the attention block to generate channel attention. The attention mechanism contains three parts: the squeeze, excitation, and attention. Firstly, the squeeze function are used as

(1) $$\begin{array}{c} F_{sq}\left(U_{c}\right)=\frac{1}{H\times W}\overset{H}{\underset{i=1}{\sum }}\overset{W}{\underset{j=1}{\sum }}U_{c}\left(i,j\right)\text{.} \end{array}$$

Making an average for the global situation, adding all eigenvalues in all channels and taking an average for them. It is essentially a calculation formula of global average pooling. And then the excitation function is

(2) $$\begin{array}{c} F_{ex}\left(z,w\right)=\partial \left(g\left(z,w\right)\right)=\partial \left(w_{2}\delta \left(w_{1}z\right)\right), \end{array}$$

where *w*_1_, *w*_2_ are dimensionality, function *∂* is the Sigmoid function, and function *δ* is ReLU.

### 2.4. Model Description

This paper aims at using the neural networks to solve the problem of the enterprise's risk credit evaluation. In recent years, the development of big data has laid a good foundation for us to RNN. The reasonable and effective enterprise's risk evaluation could reduce risks for lending institutions and improve the utilization rate of funds. It helps prevent the frequent occurrence of credit risks which lead to the enterprises bankrupt or running away and other phenomena.

**Figure 2:**
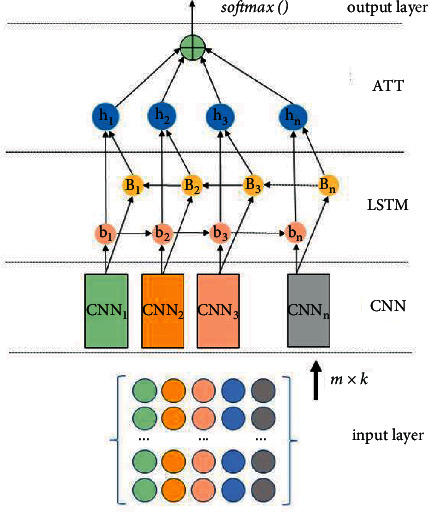
The structure of CNN-LSTM-ATT.

In this paper, the structure of the credit evaluation method based on CNN-LSTM-ATT model is presented as shown in Figure 2. Firstly, the characteristic behavior data of an enterprise is represented in the form of a matrix, and all the behavior characteristics of an enterprise are represented in the form of row vector. Then input the data to the CNN. Next, it enters the CNN layer and the LSTM layer. Taking advantages of the CNN and the both way LSTM network to maintain behavior information and extract features. Then, it enters the attention mechanism layer. Using the attention mechanism to identify the most important characteristics of enterprises behavior and measuring the characteristics of LSTM output. Finally, the final classification of the characteristic output could be obtained.

The formulas used in the CNN layer include the convolution formula and the pooling formula:

(3) $$\begin{array}{c} c^{\left(l\right)}=f\left(c^{\left(l-1\right)}⊗w+b^{\left(l-1\right)}\right)\text{,}\\ {\tilde{c}}^{\left(l\right)}=\max_{PZ}c^{\left(l\right)}\text{.} \end{array}$$

The gate formula used in LSTM layer are:

(4) $$\begin{array}{c} f_{t}=\sigma \left(w_{f}\cdot \left[h_{t-1},x_{t}\right]+b_{f}\right)\text{,}\\ i_{t}=\sigma \left(w_{i}\cdot \left[h_{t-1},x_{t}\right]+b_{i}\right)\text{,}\\ o_{t}=\sigma \left(w_{o}\cdot \left[h_{t-1},x_{t}\right]+b_{o}\right)\text{.} \end{array}$$

In the attention mechanism layer, the key special grasping problem in the enterprise is mainly solved and the feature extraction of the previous CNN-LSTM is strengthened more effectively, which improved the accuracy of prediction. The attention mechanism is used to extract the characteristics of enterprise behavior more effectively and to grasp the key points of behavioral data. The output eigenvectors of the LSTM layer are transformed into the matrix *H*. Denote the length of each vector as *n*. Use the obtained vector and the following formula to calculate the enterprise behavior:

(5) $$\begin{array}{c} M=\tan\;h\left(H\right)\text{,}\\ \alpha =soft\;\max\;\left(w^{n}M\right)\text{,}\\ r=H\alpha^{n}\text{,} \end{array}$$

where *H* ∈ *R*^*dn*^, *d* denotes the dimension of vector; *w* is the parameter vector, and *w*^*n*^ is the transposition of *w*; the dimension *w*, *α*, and *r* are corresponding to *d*, *n*, and *d*. Finally, the output of enterprise behavior for classification is as follows:

(6) $$\begin{array}{c} c^{*}=\tan\;h\left(r\right). \end{array}$$

## 3. Setting of Experiment

### 3.1. Experimental Environment

The experimental environment configuration in this paper is shown in the following Table 1:

The development language of experiment in this paper is *Python* (3.7.4) and PyCharm integrated development environment is used. The matrix operation used in the experiment is calculated by NumPy library of *Python*. The Pandas library is used for data processing and data analysis. Scikit-learn library for machine learning and library Matplotlib for drawing tool are used to accomplish the experiment. The Keras library is regarded as a deep learning tool and the TensorFlow is chosen at the back end.

### 3.2. Parameter Setting of Experiment

In terms of indexes for model evaluation, it is necessary to evaluate the default probability of enterprises. The commonly used index in the field of credit scoring: AUC (area under curve) is selected in this paper. The value of AUC is equal to the area under the receiver operating characteristic (ROC) curve. The AUC value would be larger if the area under the ROC curve is larger. Furthermore, the classification result of the model will be better. ROC curve and AUG are often used to evaluate the strengths and weaknesses of a binary classifier. The horizontal ordinate of the ROC curve is false positive rate (FPR) and the vertical ordinate is True Positive Rate (TPR). Four indexes are needed to calculate FPR and TPR : TP, TN, FP, and FN. The specific meanings are shown in Table 2.

Then the calculation formula of TPR and FPR is given as follows:

(7) $$\begin{array}{c} TPR=\frac{TP}{TP+FN}\text{,}\\ FPR=\frac{FP}{TN+FP}\text{.} \end{array}$$

Table 3 represents the parameter setting of the CNN-LSTM-ATT model.

**Figure 3:**
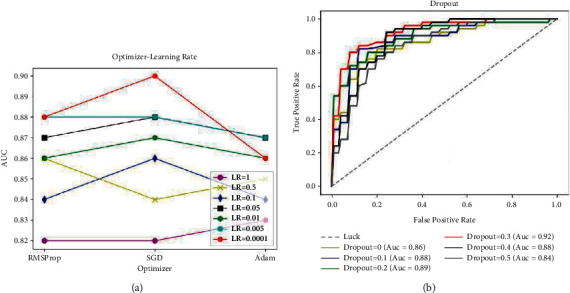
Experimental results of super-parameter setting. (a) Optimizer. (b) False positive rate.

In this paper, a super-parameter selection experiment is carried out to determine the parameter values of optimizer, learning rate, and dropout.  Figure 3 shows the schematic diagram of the results of the super-parameter selection experiment.

The result of optimization experiment is conducted on the optimizer-learning rate as Figure 3(a). When SGD is selected as the optimizer and the learning rate is 0.0001, the evaluation index achieves the optimal solution. Then make dropout selection experiment with the Optimizer = SGD, Learning Rate = 0.0001, and select Dropout as 0, 0.1, 0.2, 0.3, 0.4, and 0.5 for five times, respectively. The results are shown in Figure 3(b). When the dropout is selected as 0.3, the optimal solution of the evaluation index is obtained. In conclusion, the Optimizer = SGD, Learning Rate = 0.0001, and Dropout = 0.3.

## 4. The Experimental Comparison among Different Models

In this section, the four models of CNN, LSTM, CNN-LSTM, and CNN-LSTM-ATT are compared and tested. Firstly, the evaluation performance of the four models under different parameters is analyzed and compared, respectively. Then, a set of fixed parameters is selected for the realistic data grouping test and comparison of the four models.

### 4.1. Comparison of the Four Models under Different Parameters

**Figure 4:**
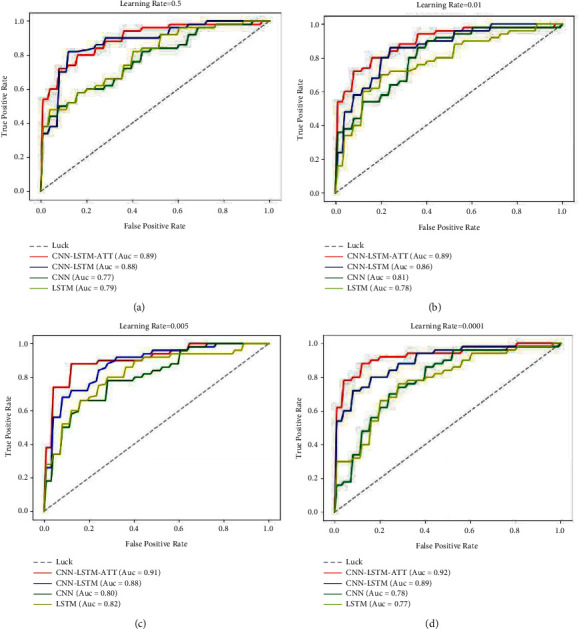
ROC curves of four models under different learning rates.

Similar to the super-parameter setting experiment, select Dropout = 0.3, Optimizer = SGD. The evaluation performance of the four models (CNN, LSTM, CNN-LSTM, and CNN-LSTM-ATT) under different LR parameters is analyzed and compared. The experimental results are shown in Figure 4. It can be seen from Figure 4 that the CNN-LSTM-ATT model used in this paper has the best result among the four models no matter which value the LR takes.

**Figure 5:**
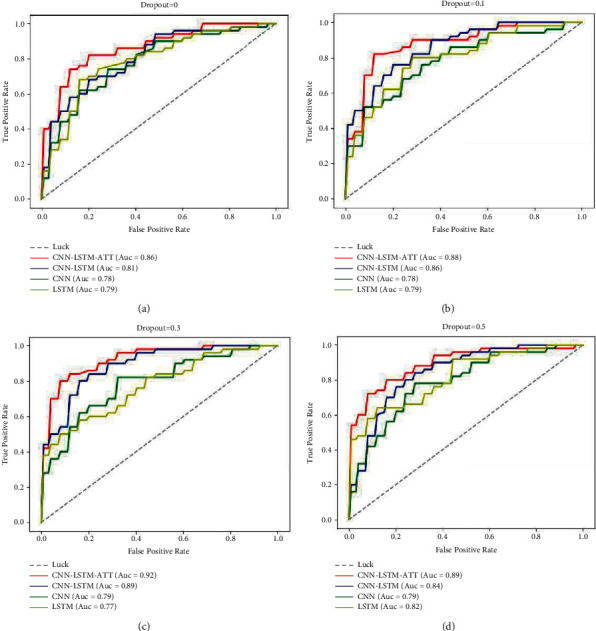
ROC curve of four models under different dropout values.

We found that the evaluation results of the four models are more comparative when the Learning Rate = 0.005. The evaluations of CNN and LSTM are near the optimal solution. Therefore, we chose Optimizer = SGD, Learning Rate = 0.005, and then made a comparative analysis experiment on dropout changes of multiple models. The experimental results are shown in Figure 5.

The results of dropout experiments with the Optimizer = SGD and the Learning Rate = 0.005 show that the CNN-LSTM-ATT model used in this paper has a better evaluation result than the other three comparison models in a variety of dropout parameter settings. Thus further illustrates the advantages of the CNN-LSTM-ATT model used in this paper in enterprise credit assessment.

### 4.2. Data Grouping for Comparison of Four Models

Before grouping test, we choose the Optimizer = SGD, Learning Rate = 0.0001, Dropout = 0.3, Optimizer = SGD, Learning Rate = 0.005, and Dropout = 0.3. Then, we randomly divide the original data sets into three experimental sets Group 0, Group 1, and Group 2, respectively. With three groups of data sets, we carry out AUC evaluation and comparison for four models CNN, LSTM, CNN-LSTM, and CNN-LSTM-ATT.

Table 4 lists the average performance of the four different models under the three experimental data sets. In each data set, the proposed CNN-LSTM-ATT model is superior to the other three models in most cases. The results show that CNN-LSTM-ATT has a good adaptive ability to the uncertainty of enterprise behavior. Compared with the original CNN-LSTM model, the attention-based model has higher accuracy and robustness. The attention module improves the AUC of the CNN-LSTM model. In most cases, the performance of proposed CNN-LSTM-ATT is better than CNN, LSTM, and CNN-LSTM models, which also reflects the advantages of the proposed space-time attention.

**Figure 6:**
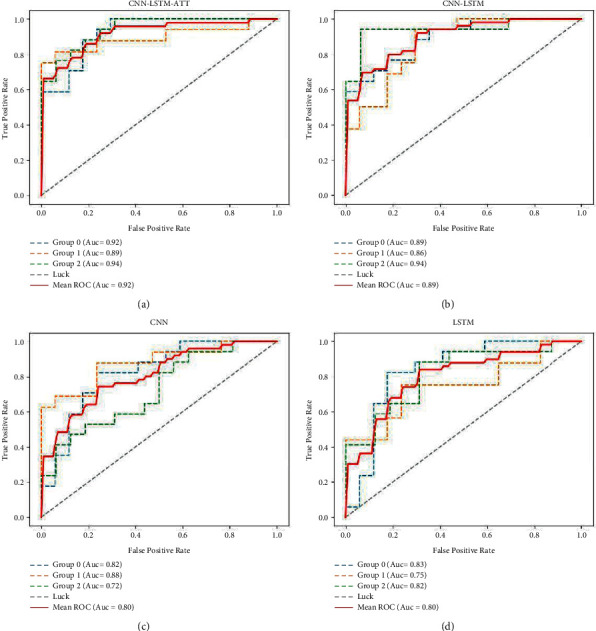
ROC curve of four models under three groups data.

AUC predicted by the model in the experiment is shown in Figure 6. In the original data set, the CNN-LSTM-ATT model proposed in this paper has the best accuracy in all three experimental sets. In the data sets of Group-0 and Group-2, the CNN-LSTM-ATT model has the best accuracy in most moments compared with the other three models. At the same time, the performance of the CNN-LSTM model is also better than that of a single CNN or LSTM model. In the Group-1 data set, the performance of the CNN-LSTM model is slightly lower than that of the CNN model, while the CNN-LSTM-ATT model remains optimal, which also indicates that the CNN-LSTM-ATT model in this paper has good robustness and accuracy. The data set has a great impact on the evaluation of the model. The training of the model with data set Group-2 has a better performance, while the average level of the training of the model with Group-1 data set is poor.

## 5. Conclusion and Future Work

In this paper, the CNN-LSTM-A TT neural network is proposed to solve the problem of enterprise's credit risk evaluation. Super-parameter selection experiment and comparison experiment of different models are conducted on the realistic data set. The validity and robustness of the CNN-LSTM-ATT model in solving the problem of credit risk evaluation are comprehensively analyzed. Super-parameter selection experiments are carried out. It found out that the classification result would be the best and the AUC value turns to 0.9 when the Optimizer = SGD and Learning Rate = 0.0001. After determining the optimizer and the learning rate, dropout selection experiment is put forward. The experiment found that the classification result would be best under Optimizer = SGD and Learning Rate = 0.0001 and the AUC value turns to 0.92 when the Dropout = 0.3. Finally, we determine the value of three super parameters, Optimizer = SGD, Learning Rate = 0.0001, and Dropout = 0.3.

In order to further evaluate the effect of the CNN-LSTM-ATT model on credit risk evaluation, we conduct a comparative analysis experiment of four different models. CNN, LSTM, and CNN-LSTM are chosen as the objects of comparison. First, a comparison experiment is carried out to compare and analyze the evaluation results of the four models by different dropout rate and learning rate. The experiment shows that the CNN-LSTM-A TT model still has the optimal performance effect among the four models under different learning rate and the CNN-LSTM-ATT model also maintains the best result under different dropout. Then, the data grouping for comparison of four models is presented. As a result, four kinds of models are all performance best under the Optimizer = SG, Dropout = 0.3, and CNN and LSTM model are relatively better under Learning Rate = 0.005. In order to guarantee the objectivity, we select two different sets of parameters respectively to ensure that the evaluation of each model is close to the best result. The classification results of four kinds of the model are tested under three separate data sets. The experimental results show that the CNN-LSTM-ATT model has the best accuracy in most moments compared with the other three models on three different data sets.

The proposed LSTM in this paper has solved the problem that RNN appears seriously gradient disappears under the long training sequence. CNN-LSTM-ATT solves the problem that traditional method LR and RF have low accuracy and the shortage of robustness when the feature space is very large. The prediction accuracy is also higher than that of the original CNN and the original LSTM, which also indicates that the CNN-LSTM-ATT model in this paper has better robustness and accuracy.

The future research work will focus on two aspects: one is to consider the impact of abrupt factors on the credit strategy of micro, small, and medium-sized enterprises and build a credit evaluation model; the second is to study the impact of affiliated enterprises on credit risk rating and construct a credit evaluation model.

## Figures and Tables

**Table 1:** The experimental environment configuration.

Experimental environment	Parameter
Operating system	Windows 10 × 64
Processor	CPU intel (*R*) core (TM) i5-9300H@2.40 GHz @2.40 GHz
Video card	GTX 1650 4G
Memory	8G
Hard disk	500G
Development language	*Python* (3.7.4)

**Table 2:** The indexes of calculating ROC.

Indexes	Meaning of indexes
TP	No default and correct prediction
TN	No default and wrong prediction
FP	No default and correct prediction
FN	No default and wrong prediction

**Table 3:** The parameter setting of the CNN-LSTM-ATT model.

Parameter	Meaning of parameter	Value of parameter
time_series	The step length	20
behavior_num	Number of behavioral indexes	14
iB-LSTM_units	Number of neurons for LSTM	64
Kernel_size1	The size of the convolution kernel for convolution layer 1	1 × 3
Kernel_size1	The size of the convolution kernel for convolution layer 2	3 × 3
Pool	Pooling method	Max
Stride	Length of pooling or convolutional	1
Dropout	Rate of LSTM dropout	0.3
batch_size	The size of batch	128
Epoch	Number of iterations	20
Optimizer	The optimizer	SGD
Learning rate	The learning rate	0.0001

**Table 4:** The AUC of different models under three groups data.

Model	Group-0	Group-1	Group-2	Mean
**CNN-LSTM-ATT**	**0.92**	**0.89**	**0.94**	**0.92**
CNN-LSTM	0.89	0.86	0.94	0.89
CNN	0.82	0.88	0.72	0.80
LSTM	0.83	0.75	0.82	0.80

## Data Availability

The data used to support the findings of this study are available from the corresponding author.
